# Single-Cell Landscape of the Cochlea Revealed Cell-Type-Specific Diversification in *Hipposideros armiger* Based on PacBio Long-Read Sequencing

**DOI:** 10.3390/biom15020211

**Published:** 2025-02-01

**Authors:** Mingyue Bao, Xue Wang, Xintong Li, Ruyi Sun, Zhiqiang Wang, Tinglei Jiang, Hui Wang, Jiang Feng

**Affiliations:** 1 College of Life Science, Jilin Agricultural University, Changchun 130118, China; baomingyue@mails.jlau.edu.cn (M.B.);; 2 Jilin Provincial International Cooperation Key Laboratory for Biological Control of Agricultural Pests, Changchun 130118, China; 3 Jilin Provincial Key Laboratory of Animal Resource Conservation and Utilization, Northeast Normal University, Changchun 130117, China

**Keywords:** bat, cochlea, snRNA-seq, high-frequency hearing, SGN

## Abstract

Echolocation represents one of the most rapid adaptive sensorimotor modulation behaviors observed in mammals, establishing bats as one of the most evolutionarily successful mammals. Bats rely on high-frequency hearing for survival, but our understanding of its cellular molecular basis is scattered and segmented. Herein, we constructed the first single-cell transcriptomic landscape of the cochlea in *Hipposideros armiger*, a CF-FM bat, using a PacBio-optimized genome and compared it with the results obtained from unoptimized original genomes. Sixteen distinct cell types were distributed across five spatial regions of the cochlea. Notably, through hematoxylin and eosin staining and fluorescence in situ hybridization, we identified new types of spiral ganglion neuron (SGN) cells in the cochlea of *H. armiger*. These SGN cells are likely critical for auditory perception and may have driven the adaptive evolution of high-frequency hearing in this species. Furthermore, we uncovered the differentiation relationships of among specific cell types, such as the transition from supporting cells to hair cells. Using the cochlear cell atlas as a reference, cell types susceptible to deafness-associated genes (in the human) were also identified. In summary, this study provides novel insights into the cellular and molecular mechanisms underlying the adaptive high-frequency hearing in bats and highlights potential candidate cell types and genes for therapeutic interventions in hearing loss.

## 1. Introduction

Since the discovery of bat echolocation behavior, it has attracted considerable interest from researchers, aiming to explore the diversity of ways in which animals perceive the world. Unlike most mammals that rely on vision or smell for directional information, bats have developed a unique mode to perceive surrounding environment. They primarily depend on advanced echolocation for flight navigation [1,2], prey tracking and positioning [3,4], and group communication [5,6]. Echolocation enables bats to accurately analyze spatial details during flight, such as detecting the shape, size, and texture of objects [7,8,9]. Hearing is integral to echolocation for bats, as it is essential for maintaining a stable vocalization pattern. Consequently, bats have evolved high-frequency hearing that are highly attuned to echolocation acoustic signals, such as those of *Cloeotis percivali*, which range from 217 kHz to 225 kHz [10].

Adaptive changes of high-frequency hearing in bats run through the entire auditory system from the peripheral auditory organs to meet the needs of echolocation. Sound waves are transmitted from the outer ear following by a series of mechanical vibrations and conduction processes. As a crucial component of the peripheral auditory system, the cochlea converts sound waves into electrical signals through hair cells (HCs), which are then transmitted to the central nervous system of the brain, ultimately achieving auditory perception [11]. The cochlear spirals of bats are 2.5 to 3.5 turns along the modiolus, whereas only approximately 1.75 turns in the cochleae of primates [12,13,14]. Bats that use CF-FM and frequency-modulated (FM) acoustic signals for echolocation possess larger cochleae with more turns compared to rodents like rats and mice, which may be related to their distinct echolocation behaviors [15]. In CF-FM bats, the CF component of the second harmonic of the echolocation signals, also known as the dominant frequency, and its adjacent frequencies, are excessively characterized, forming a specialized region known as the “auditory fovea” [16,17,18]. Neurons in this region exhibit high tolerance to sound intensity and sharp frequency tuning, allowing them to accurately sense and adjust to Doppler frequency shifts and lock onto periodic frequency changes produced by insect wing vibrations [19]. Recently, a neuroanatomical study revealed differences in the structure of Rosenthal’s canal in the cochlea of bats with different echolocation types, suggesting it may be an evolutionary driving factor behind bats’ diverse echolocation strategies [16]. This finding significantly enhances our understanding of the basic morphological structure of the inner ear in echolocating bats.

In the past two decades, researchers have increasingly explored the molecular bases and evolutionary mechanisms of high-frequency hearing in echolocating bats at the genetic level. Studies have demonstrated that the formation and implementation of high-frequency hearing may rely on a class of highly expressed genes [20,21,22,23], particularly those related to nervous system activity in the cochlea, which may promote high-frequency auditory perception in echolocating bats. Additionally, several hearing-related genes, including *Prestin*, *KCNQ4*, *Tmc1*, and *Pjvk* [24,25,26], have been reported to be undergo convergent or parallel evolution. *Prestin* and *KCNQ4*, in particular, have been identified in positively selected [27,28] sites in laryngeal echolocating bats, indicating their crucial role in the adaptive evolution of high-frequency hearing. Despite our knowledge of these adaptively evolved hearing genes, the precise cellular locations of the key auditory genes within the bats’ cochleae are still not clear. The sources and specific locations of important genes, the origin and differentiation relationships between various cells, and the functional roles of intracellular gene actions remain to be elucidated.

In recent years, single-cell RNA-sequencing (scRNA-seq) has been proven to be one of the transformative technologies in the life sciences, revolutionizing molecular and cellular biology, rapidly enhancing our ability to elucidate the cochlear cell type, state, origin, and differentiation of echolocating bats [29]. Taking advantages of the complete structure of the full-length transcriptome in improving genome annotation, this study aims to utilize the genome optimized by the PacBio full-length transcriptome as a reference to analyze adult bat’s cochlea using single-nuclei RNA sequencing (snRNA-seq) for the first time. This approach seeks to reveal a comprehensive cochlear cell atlas of bats, including specific neurons in the CF-FM bat cochlea driving the adaptive evolution of high-frequency hearing. Potential differentiation relationships and differentiation genes between auditory cell types were elucidated. By exploring key cell types and molecular mechanisms underlying high-frequency hearing in echolocating bats, this study provides a significant reference for research into high-frequency hearing in echolocating mammals. Notably, several positively selected genes identified in echolocating bats are associated with human hearing loss. In-depth cellular localization and association analyses of these genes could offer new insights for gene therapy targeting the inner ear in patients with hearing loss.

## 2. Materials and Methods

### 2.1. Sample Collection and RNA Preparation

To minimize individual variation and sex-related differences, six adult male *H. armiger* (CF-FM bats), each weighing 66.0 ± 1.0 g, were collected from Feilong Cave in Xingyi City, Guizhou Province, China (24°58′ N, 104°52′ E) on 5 July 2021. Two individuals were used for full-length transcriptome sequencing, while the remaining four bats (eight cochleae) were used for nuclear extraction (snRNA-seq). To ensure that single-cell sequencing could capture a broader range of cochlear cell types, the samples collected included the entire cochlear tissue, encompassing both the bone capsule and all enclosed soft tissues, thereby preserving the diversity and integrity of cochlear cell types.

After carefully detaching the cochlea from the temporal bone, we gently excised the surrounding structures using ophthalmic surgical scissors and forceps, leaving only the spiral-shaped cochlear tissue encased within the bony structure. Bilateral cochlear tissues were harvested, immediately rinsed with DEPC-treated water (pre-cooled to 4 °C), transferred into RNase-free freezing tubes, and then snap-frozen overnight in liquid nitrogen. The samples were stored in an ultra-low temperature freezer at −80 °C until the next experimental stage.

### 2.2. SnRNA-Seq Library Preparation and Sequencing

To capture the transcriptome of individual nuclei, we utilized the Chromium Single Cell 3′ Reagent V3 Kit (10X Genomics, Pleasanton, CA, USA). Nuclei were loaded onto a 10X Genomics GemCode single-nucleus instrument to generate single-nucleus gel bead-on-emulsion (GEM). Reverse transcription and library preparation were performed using the Chromium Next GEM Single Nucleus 3′ Reagent Kits v3.1 (10X Genomics, CG000204), following the manufacturer’s instructions. The cDNA library was sequenced on the Illumina platform with 150-bp paired-end reads (Genedenovo Biotechnology, Guangzhou, China).

### 2.3. Quality Control and Expression Quantification

In a previous study, we analyzed five tissue organs (brain, bilateral cochlea, heart, liver, and muscle) of adult *H. armiger* using PacBio single-molecule real-time (SMRT) sequencing. The first full-length transcriptome map of *H. armiger* was obtained, existing genome annotations were optimized and updated, and novel genes were identified [30]. The original genome of *H. armiger*, released in 2016 (ncbi_GCF_001890085.1) [31], and the genome with optimized annotation by PacBio (ncbi_SRR22307099) were used as references. The official 10x Genomics analysis software, Cell Ranger (version 3.1.0), was employed to perform preliminary statistics on the number of reads obtained and the sequencing quality of each sample. To further investigate the cell types in the cochlea of CF-FM bat at the single-cell level and assess the applicability of the two aforementioned reference genomes, we conducted a series of analyses. After filtering and correcting barcodes and unique molecular identifiers (UMIs) in Cell Ranger, unfiltered feature barcode matrices were obtained to identify and distinguish cells from non-cells, leading to the identification of effective cells. Finally, gene expression levels were quantified using the number of UMIs to obtain quantitative results of cell-gene expression.

### 2.4. SnRNA-Seq Data Analysis and Cell-Type Identification

Further cell filtering, normalization, cell clustering, and analysis of differentially expressed genes in cell clusters with marker genes screening were performed using the Seurat package (version 3.1.1) [32] in R software (version 3.6.3). DoubletFinder (version 2.0.3) [33] was used to calculate the probability of GEM being multicellular (pANN value), determine the multicellular filtering threshold for each sample, and sequentially perform multicellular filtering. After removing low-quality cells, data consolidation and batch effect correction were conducted using Harmony [34]. The dimensionality-reduced data were clustered using the soft k-means clustering algorithm, which assigns cells probabilistically to clusters to maximize the diversity of the dataset within each cluster. The global center of all datasets within each cluster and the center of each specific dataset were calculated. Based on these centers, correction factors for each dataset were determined, and cells were adjusted to cluster towards the center. These steps were iteratively repeated until the clustering effect stabilized.

Next, t-distributed stochastic neighbor embedding (t-SNE) and uniform manifold approximation and projection (UMAP) clustering methods were employed to visualize the results of single-cell cluster classification and to generate a preliminary drawing of s single-cell atlas of *H. armiger* cochlea. Differential gene expression analysis across cell clusters was performed using the rank-sum test in Seurat to identify genes upregulated in specific clusters. The screening criteria were as follows: (1) genes expressed in more than 25% of cells in the target cluster; (2) *p* ≤ 0.01 and log_2_FC ≥ 0.36. Marker genes were selected from the top 50 genes with the highest upregulated in each cluster. We also identified marker genes and cell types in conjunction with previously published research and mapped the two sets of *H. armiger* cochlea cells before and after optimization by PacBio. Marker genes and their expression levels for each cell type are presented in Table S1.

### 2.5. Determination of Intercellular Differentiation Relationship

PAGA software [35] was utilized to temporally arrange each cell, reflecting the differentiation relationships between cells. All cell scatters were simplified into topological networks to visualize the relationships between different cell clusters in the cochlea. Pseudotime trajectory analysis was performed using the monocle package (version 3.0) [36] to elucidate similarities and differentiation relationships between cell clusters of interest. The gene expression matrix generated by 10x Genomics was imported into monocle 2, and cell trajectories were visualized for various differentiation states and cell clusters, respectively. The dynamic trend of gene expression along branching trajectories was displayed in a heatmap. All cells were arranged on a pseudotime axis according to their developmental stages, simulating the process of cell differentiation during development. The gene selection criteria were log2FC ≥ 0.36 and *p*-value ≤ 0.01. Differentially expressed genes from different cell trajectories were categorized. GO [37] and KEGG [38] analyses were separately performed for differentiation fate differential genes in each module. The calculated *p*-values were corrected for false discovery rate (FDR), using FDR ≤ 0.05 as the threshold. This analysis provided insights into life development, such as dynamic changes in gene expression during cell differentiation and the different differentiation fates of progenitor cells, aiming to reveal a more detailed origin and differentiation trajectory of cells in the bat cochlea.

Weighted correlation network analysis (WGCNA) was performed on all cell types to identify gene modules significantly associated with cell types using WGCNA (version 1.70-3) package [39] embedded in R software. After removing low-quality genes, hierarchical clustering of all samples was conducted based on gene expression levels to construct gene clustering trees and define gene modules. Modules with similar expression patterns were merged to obtain dynamic module clusters. A heatmap of cell cluster expression patterns was generated using module eigenvalues to display the expression patterns of module genes in each cell type. For the modules significantly associated with each cell type, the top 500 genes with the highest weight values were selected, and a gene interaction regulatory network was constructed using Cytoscape (version 3.10.1). Additionally, given the critical role of TFs in transcriptional regulation, the predicted protein sequences were compared with the animal TF database (TFdb) [40] via hmmscan. Key TFs related to specific cell types in each module were identified to further elucidate the regulatory mechanisms of intracellular TFs of interest.

### 2.6. Hematoxylin and Eosin Staining

To visualize the detailed cellular architecture of *H. armiger* cochlea, the tissue was stained with hematoxylin and eosin. First, the cochlear tissue was removed and immersed in tissue fixative (Servicebio, Wuhan, China, G1101) for overnight fixation. The tissue was then decalcified by placing it in EDTA decalcification solution (Servicebio, G1105) in a sealed constant temperature shaker, with the decalcification solution replaced every 2–3 days. The softened cochlea was then rinsed under running water, dehydrated with sequential gradients of alcohol, and dipped in wax for embedding after xylene transparency.

The paraffin-embedded tissue blocks were sectioned to a thickness of 4 μm using a microtome. The sections were dewaxed, rinsed briefly under running water, and treated with high-definition constant dyeing pre-treatment solution for one minute. Following this, the sections were stained with hematoxylin for 3–5 min, rinsed with running water, and subjected to a differentiation solution to achieve appropriate staining intensity. The sections were then rinsed with PBS, followed by a bluing step with a blue-return solution, and further rinsed with running water. Subsequently, the sections were dehydrated in 95% ethanol for one minute, stained with eosin solution for 15 s, and then subjected to a series of dehydration and clearing steps, as follows: 2 min in anhydrous ethanol I, 2 min in anhydrous ethanol II, 2 min in anhydrous ethanol III, 2 min in n-butanol I, 2 min in n-butanol II, 2 min in xylene I, and 2 min in xylene II. Finally, the sections were mounted with neutral gum and allowed to dry.

### 2.7. RNA In Situ Hybridization

To elucidate the expression of marker genes in the selected key cell types of the cochlea, FISH was employed for validation. Following the removal of the cochlea from one side of the *H. armiger*, the tissue was rinsed with PBS, immediately fixed in animal in situ hybridization fixative (Servicebio, G1113) for over 12 h, and then stored at 4 °C. After fixation, a tissue block, approximately 3 mm thick, was excised from the target area under a fume hood. FISH probes were designed and synthesized by Servicebio (Wuhan, China). The tissue block was then dehydrated using an alcohol gradient, cleared with xylene, and embedded in paraffin wax. Tissue paraffin blocks were sectioned into 4 μm thick slices and then oven-baked at 62 °C for two hours. The sections were then sequentially placed in dewaxing solution I for 15 min, dewaxing solution II for 15 min, anhydrous ethanol I for five minutes, anhydrous ethanol II for five minutes, 85% ethanol for five minutes, and 75% ethanol for five minutes, followed by soaking in DEPC water. After cooling to room temperature, proteinase K (20 μg/mL) was added dropwise, and the sections were digested at 37 °C. The sections were then rinsed with distilled water and PBS three times for five minutes each. Prehybridization solution was added dropwise and incubated at 37 °C for one hour. The prehybridization solution was then removed, and the hybridization solution containing the probe was added dropwise. The sections were hybridized overnight in a thermostat. Following hybridization, the sections were washed sequentially with 2 × SSC at 40 °C for 10 min, 1 × SSC at 40 °C for 10 min, and 0.5 × SSC at room temperature for 10 min. 6-diamidino-2-phenylindole (DAPI) staining solution was added dropwise and incubated for eight minutes in the dark. The sections were then rinsed and sealed with anti-fluorescence quenching sealer. Finally, the stained sections were observed and imaged using a Nikon laser confocal microscope (NIKON Eclipse Ti). All reagents, instruments, and RNA in situ hybridization experiments mentioned were conducted in an RNase-free environment following DEPC treatment.

### 2.8. Localization of Hearing Loss Genes and Positively Selected Genes

Genes associated with human hearing loss were identified from the cochlear snRNA-Seq dataset of *H. armiger*, using gene databases such as the Hereditary Hearing Loss Homepage (https://hereditaryhearingloss.org/, accessed on 6 May 2024) and relevant studies by Korrapati et al. [41,42,43] from 2019–2023. The nonsynonymous-to-synonymous-rate ratio (ω), based on the amino acid sequences of different species, was used to indicate changes in selective pressures during molecular evolution. A ratio of ω > 1 signifies that the gene is under positive selection, suggesting that mutations at these loci are advantageous and become fixed over generations. Key hearing genes, especially those critical in echolocating species and genes under positive selection in adaptative evolution, were mapped in the *H. armiger* cochlear atlas [20,21,24,25,27,44,45,46,47,48,49,50,51,52] to identify the cell types expressing these genes. Cluster averages of *z*-score-adjusted normalized gene expression were calculated, and heatmaps were used to visualize the *z*-scores of these cluster averages.

## 3. Results

### 3.1. PacBio-Optimized Single-Cell Results Facilitate More Precise Cell Type Definition

**Figure 1:**
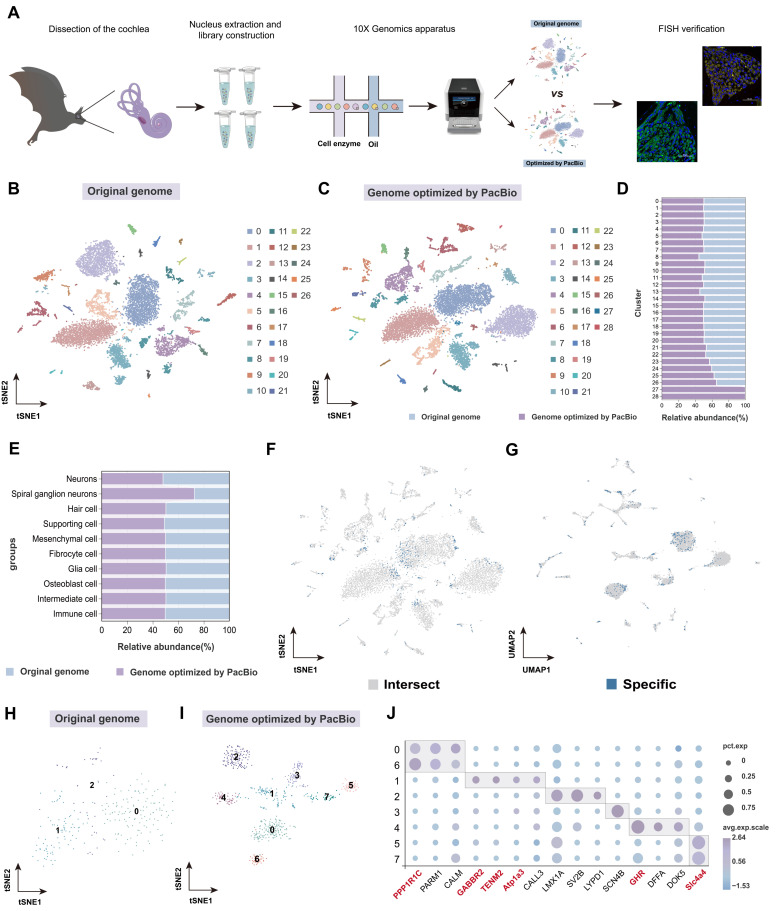
Cochlear cell expression atlas of *H. armiger* before and after PacBio optimization. (**A**) Schematic representation of the experimental workflow. (**B**) t-SNE visualization of 27 cell clusters based on original genome. Each dot denotes a single cell. (**C**) t-SNE visualization for 29 cell clusters based on PacBio optimized genome. (**D**) Proportion of cell numbers for each cluster with two genomes as reference. (**E**) Comparisons of cell clusters before and after optimization. (**F**,**G**) t-SNE and UMAP mapping of intersecting and specific cells after optimization. Gray and blue represent intersecting and specific cell barcodes before and after optimization, respectively. (**H**,**I**) t-SNE visualizations of re-clustered SGN cells before and after optimization, respectively. The numbers in the figure represent corresponding clusters. (**J**) Expression patterns of representative marker genes in SGN. Red font represents novel genes identified after PacBio optimization.

Here, we conducted snRNA-seq analysis of the cochlea in *H. armiger* using both the original genome and the genome optimized by PacBio as references (Figure 1A). The results optimized by PacBio and those using the original genome as a reference were shown in Table S2. The confidence of reads mapped to the transcriptome increased from 63.10% before optimization to 67.50% after optimization. This improvement is likely due to the ability of full-length transcriptomics to identify new transcripts missing from existing genome annotations. Overall, the data obtained from both references were sufficient to support subsequent analyses. Visualization of cell cluster classification using t-SNE (Figure 1B,C) and UMAP (Figure S1A,B) demonstrated that the single-cell results optimized by PacBio full-length transcriptome detected two more clusters than those before optimization at the same resolution (Figure 1D).

The results before and after optimization were classified based on the same marker genes. It was found that the percentage of optimized SGN cells (72.60%) was significantly higher than that before optimization (27.40%) (Figure 1E). The intersecting cells (gray) and specific cells (blue) from the two single-cell datasets, before and after genome optimization, are presented in Figure 1F,G. The results showed that the specific cells obtained after optimization were mainly distributed in neuronal clusters (cluster 0) and SGN-related clusters. All SGN cells, both before and after PacBio optimization, were further subdivided into clusters at the same resolution. The SGN cells before optimization were only able to be divided into three clusters (Figure 1H), with sparse and dispersed cell arrangements. In contrast, the PacBio-optimized SGN cells were re-clustered into eight clusters (Figure 1I), twice as many as before optimization, with cells arranged in tight clusters.

Finally, the distribution of SGN marker genes (Figure 1J) showed that most of the marker genes defining SGN clusters were novel genes identified through optimization (red), indicating that the optimized genome enhanced the accuracy of SGN definition. When mapping these novel genes to five major cell categories (Figure S1C), the highest proportion was found in neuronal cell clusters. This demonstrates that using the PacBio-optimized *H. armiger* genome for single-cell analysis could help us define cell types more accurately and, in particular, improve the definition of SGN cells. Therefore, the optimized single-cell data were used for subsequent analyses for in-depth exploration.

### 3.2. New Types of SGN Cells in the Cochlea of H. armiger

**Figure 2:**
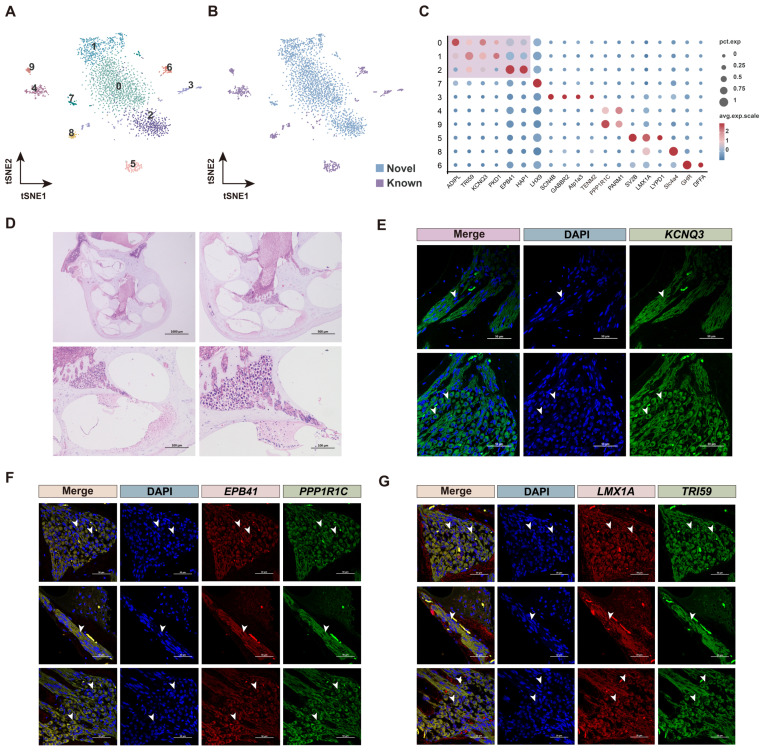
Re-cluster of all neurons. (**A**) t-SNE visualization for re-cluster of all neurons. The numbers in the figure represent corresponding clusters. (**B**) All neurons categorized into novel neurons and known neurons. (**C**) Expression patterns of cluster-specific marker genes. Size of the dot represents the percentage of specific marker genes expressed in every cluster (corresponding with pct. exp.), and dot color represents the average scaled expression (avg. exp. scale). (**D**) HE staining of *H. armiger* cochlear structures. (**E**) FISH results of *KCNQ3* (green) with cell nuclei stained using DAPI (blue) in cochlear SGN cells. (**F**) *EPB41* (red) and *PPP1R1C* (green) in cochlear SGN cells with nuclei stained using DAPI (blue) by FISH. (**G**) *LMX1A* (red) and *TRI59* (green) in SGN cells with nuclei stained using DAPI (blue) by FISH. All fluorescent regions indicated by arrows in Figure 2E–G represent the locations of SGNs in the cochlea of *H. armiger*.

In addition to SGN cells, another type of neuronal cells obtained through PacBio optimization were distributed in cluster 0 (Figure 1C). To confirm the heterogeneity of the two neuronal cells, the cluster 0 and all SGN clusters (cluster 12,15,20,23,24,26,27) (Figure 1C) were re-clustered (Figure 2A). Clusters 3, 4, 5, 6, and 7 were defined as SGN cells using classic marker genes such as *Lmx1a*, *Slc4a4*, *Lypd1*, *SV2B*, and *SCN4B* [53,54,55,56,57] after clustering, called known neurons (Figure 2B,C). However, clusters 0, 1, and 2 in the center of the atlas remained undefined based on existing marker genes, suggesting that they are special SGN cell types in the cochlea of *H. armiger*, called novel neurons (Figure 2B,C).

Hematoxylin and eosin (H&E) staining and fluorescence in situ hybridization (FISH) results confirmed that the novel neurons (clusters 0, 1, 2) may be new SGN cell types in the cochlea of *H. armiger*. Using the known SGN marker genes *LMX1A* [53] and *PPP1R1C* [58], FISH results revealed that the novel neuron marker genes *KCNQ3*, *TRI59*, and *EPB41* were localized to the spatial position where SGNs were found (Figure 2D–G). This suggests that these novel neurons may be new types of SGN cells in the cochlea of *H. armiger*, distinct from those identified in previous studies, and could serve as reference marker genes for future studies on cochlear SGNs in bats. Furthermore, the map indicates that the new types of SGN cells encompass three types, potentially representing different subtypes. More detailed clustering results are provided in Figure S2A–C.

### 3.3. Cochlear Cell Atlas in H. armiger

**Figure 3:**
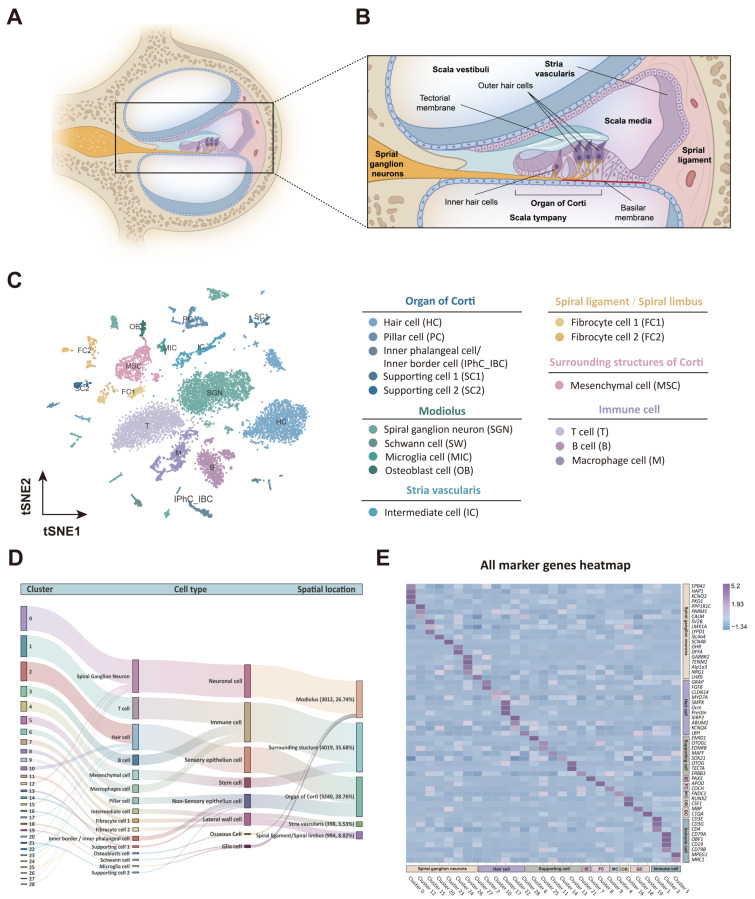
Single-cell transcriptome landscape of the cochlea in *H. armiger*. (**A**) Cross-section of the cochlear duct. (**B**) Local magnification of the cochlear cross-section. Illustrations of three fluid-filled chambers (scala vestibuli, scala media, and scala tympany) and multiple cellular structures. (**A**,**B**) were created using Biorender.com. (**C**) Left: t-SNE plot showing the distributions of different cell types in the cochlea; right: the annotation of different cell types. HC, hair cell; PC, pillar cell; IPhC_IBC, inner phalangeal cell/inner border cell; SC1, supporting cell 1; SC2, supporting cell 2; SGN, spiral ganglion neuron; SW, Schwann cell; MIC, microglia cell; OB, osteoblast; IC, intermediate cell; FC1, fibrocyte 1; FC2, fibrocyte 2; MSC, mesenchymal cell; T, T cell; B, B cell; M, macrophage. (**D**) Sankey diagram of cochlear cells categorized by clusters. (**E**) Heatmap showing the gene expression signatures of all clusters in the cochlea.

Taken together, a total of 16 cell types distributed across five major regions in the cochlea of *H. armiger* were identified using gene expression profiling and specific marker genes. These regions include cells located in and around the organ of Corti, modiolus, stria vascularis, spiral ligament, and spiral limbus, as well as various types of immune cells (Figure 3A,B). Cells localized in the organ of Corti included hair cells (HC, *GRAP*, *FGF8*, *CLD16*, *MYO7A*, *Prestin*, *Ocm*, *XIRP2*, *KCNQ4*, *ABLIM2*, and *LBH* [41,59,60,61,62,63,64,65,66,67]), pillar cells (PC, *EMID1*, and *EDNRB* [41,42]), inner phalangeal cells/inner border cells (IPhC_IBC, *MAFF*, and *SOX21* [41,68]), supporting cell 1 (SC1, *OTOG* [69], and *TECTA*1 [70]), and supporting cell 2 (SC2, *ERBB3* [71]). Cells in the modiolus comprised four major cell types: spiral ganglion neuron (SGN, *HAP1*, *KCNQ3*, *PKD1*, *EPB41*, *PPP1R1C*, *LMX1A*, *Lypd1*, *Slc4a4*, *Gabbr2*, *Atp1a3*, *NRG1*, *TENM2* [53,54,58,70,71,72,73,74,75], and so on), Schwann cells (SC and *MBP* [76]), microglia cells (MIC and *C1QA* [77]), and osteoblast cells (OB, *Runx2*, and *CSF1* [42,78]). Cells in the Stria vascularis (SV) were predominantly intermediate cells (IC and *PAX3* [43]). Other cell types included mesenchymal cells (MSC, *FNDC1* [79]) in the surrounding structure of the Corti, as well as various immune cell types (T cells: *CD3E, CD3G*, and *CD4* [80,81]; B cells: *OBF1*, *CD79A*, *CD19*, and *CD79B* [82,83,84]; and macrophage cells: *MPEG1* and *MRC1* [85,86]) (Figure 3C). The cell classification of each cluster is shown in Figure 3D, and the corresponding marker genes for each cell type are presented in Figure 3E and Table S1.

**Figure 4:**
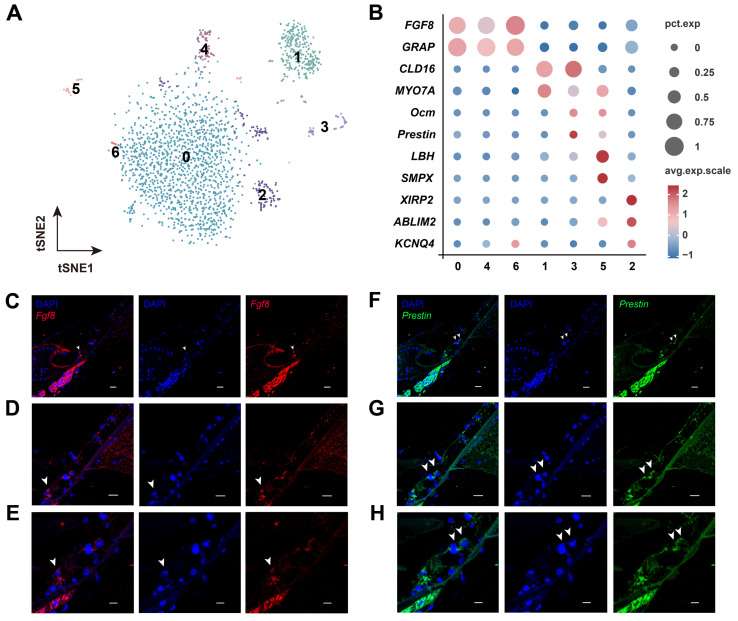
Identification of HC subpopulations. (**A**) t-SNE plot showing the distribution of subclusters of HCs. The numbers in the figure represent corresponding clusters. (**B**) Expression patterns of cluster-specific marker genes. Size of the dot represents the percentage of specific marker genes expressed in every cluster (corresponding with pct. exp.), and dot color represents the average scaled expression (avg. exp. scale). (**C**–**E**) FISH results of *Fgf8* (red) with cell nuclei stained using DAPI (blue) in cochlear IHC cells. (**F**–**H**) *Prestin* (green) in OHC cells with nuclei stained using DAPI (blue) by FISH. (**C**,**D**,**F**,**G**) scale bars: 50 μm. (**E**,**H**) scale bars: 20 μm. All fluorescent regions indicated by arrows in Figure 4C–H represent the locations of IHCs and OHCs in the cochlea of *H. armiger*.

Furthermore, our detailed analysis also identified HC subpopulations based on their canonical marker genes and unique functions (Figure 4A). For example, IHCs and OHCs were distinguished by the former expressing classical marker genes *Fgf8*, and the latter expressing *Ocm, Prestin*, *SMPX*, *XIRP2*, and *Kcnq4*, and so on (Figure 4B). We further validated *Fgf8* and *Prestin* as specifically expressed within HCs by FISH (Figure 4C–F). Collectively, we established a comprehensive cellular and molecular taxonomy of the adult cochlea of *H. armiger* (CF-FM bat), serving as a foundation for high-frequency hearing analysis.

### 3.4. Potential Differentiation Relationship Between SCs and HCs in Cochlea

**Figure 5:**
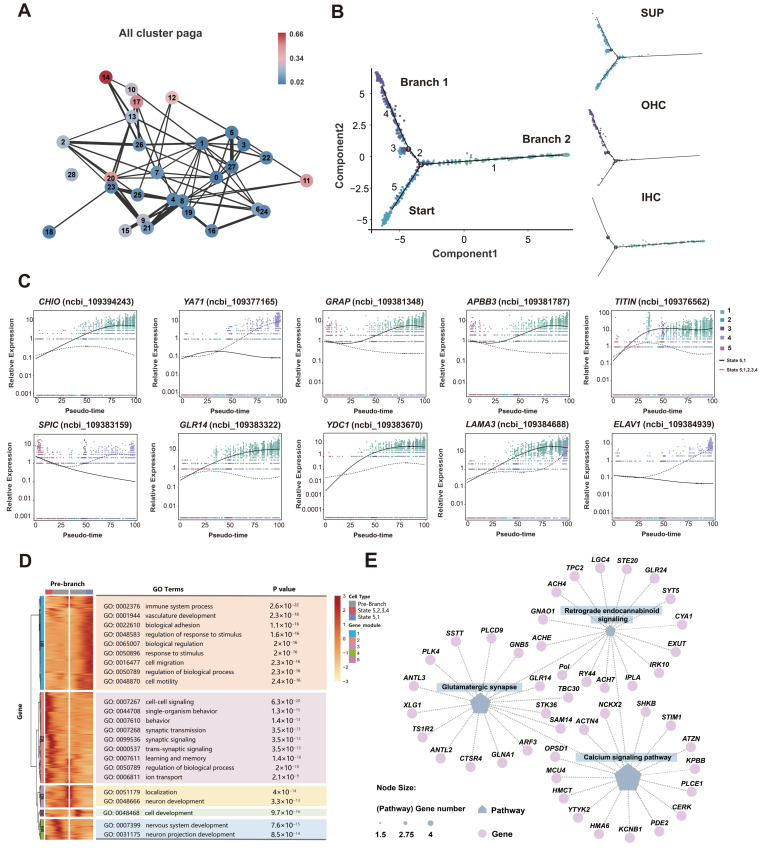
Differentiation trajectory of the SCs and HCs. (**A**) PAGA trajectory analysis of 29 clusters. Each node number represents to its corresponding cluster, and the line weight represents the statistical measure of connectivity between nodes. (**B**) Pseudotime trajectory of SCs, IHCs, and OHCs development. The numbers 1–5 on the branches represent different differentiation fate trajectories. Each dot represents a single cell. (**C**) Pseudotime expression trajectories of the top 10 significantly differential genes dependent on branch. (**D**) Trends in gene expression with branching in cells based on different differentiation fates. Significantly enriched GO terms in each cluster are shown on the right. M1–M5 represent gene modules 1–5. (**E**) The regulatory network of significant pathways and genes involved in M1–M5, with 15 genes listed for each pathway.

Partition-based graph abstraction (PAGA) analysis [35] was performed on 29 clusters (Figure 5A), revealing a potential differentiation relationship between SCs, HCs, and SGNs (clusters 10, 11, 12, 13, 14, 17, and 20). Furthermore, pseudotime trajectory analysis provided a more detailed differentiation route between SCs and HCs. SCs were identified as the starting point of developmental trajectories, ultimately differentiated into OHCs (Branch 1) and IHCs (Branch 2) (Figure 5B). During the differentiation process, some SCs differentiate into IHCs (branch 2), while others temporarily function as OHCs before fully transforming into OHCs. To differentiate between Branches 1 and 2, the marker genes expression levels of SCs, OHCs, and IHCs were labeled in the corresponding branches (Figure S3A–H). The marker genes *VIT5*, *OTOGL*, and *EDNRB* of SCs were expressed in the starting branch and middle segment of Branch 1 (Figure S3A–C), indicating their involvement in cell development and differentiation of HCs and representing the cellular distribution of SCs on the pseudotime line. Marker genes for IHCs, *FGF8*, and *GRAP* were predominantly expressed in Branch 2 (Figure S3D,E), whereas *Prestin* and *CLD16* of OHCs were primarily expressed in the middle and end of Branch 1 (Figure S3F–H), indicating the distribution locations of IHCs and OHCs on the pseudotime line, respectively. Additionally, the top 10 significantly differential genes involved in the differentiation fate from SCs to HCs are shown in Figure 5C, which may play a role in the differentiation of IHCs and OHCs from SCs in the cochlea.

The expression levels of all differential genes related to the differentiation fate of OHCs (Branch 1) and IHCs (Branch 2) are shown in a heatmap (Figure 5D) and classified into five modules (M1-M5) according to their expression profiles (Table S3). Genes from M1 and M2 were extremely highly expressed in cells at the ends of Branches 2 and 1, respectively, suggesting that these genes are involved in the differentiation of SCs into different types of HCs. Genes from M3 were highly expressed in pre-branch cells, indicating a role in maintaining the undifferentiated state of SCs or preparing them for the differentiation and development stage. Genes from M4 and M5 were more highly expressed in cells in the transition state, implying a function in the differentiation of IHCs or OHCs.

GO analysis of genes from M1–M5 revealed distinct enrichment patterns. Genes in M1 were mainly enriched in the immune system process (GO:0002376) and vasculature development (GO:0001944), related to the growth of differentiated IHCs (Figure 5D). In contrast, genes in M2 were enriched in synaptic transmission (GO:0007268) and trans-synaptic signaling (GO:0099537) in OHCs. Genes from M3, M4, and M5 were primarily involved in neuron development (GO: 0048666), cell development (GO: 0048468), and nervous system development (GO: 0007399) (Figure 5D). These results indicated that genes from different modules were enriched in specific biological processes, suggesting that strictly temporal and spatial regulation of gene expression determined the direction of SC differentiation in the cochlea. Based on the pathway and gene regulation network of M1–M5, the glutamic synapses (ko04724) and calcium signaling pathway (ko04020) show strong connectivity across all pathways (Figure 5E; Table S4). This implies their crucial role in differentiating SCs into HCs, leading to distinct outcomes for SCs during differentiation. Weight network analysis was performed to evaluate the genes in the correlation module, identifying hub genes for each cell type (Figure S4A–E), which may then be utilized as candidate genes for further correlation analysis.

### 3.5. Runx2, a Key TF in Cochlear Osteoblast Differentiation in Bats

**Figure 6:**
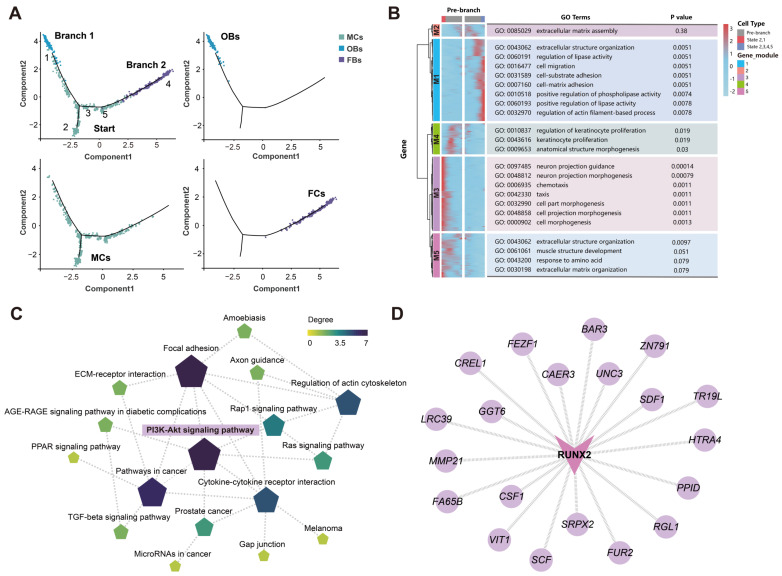
Reconstruction of the developmental trajectories of MCs, OBs, and FCs. (**A**) Pseudotime trajectory of MCs, OBs, and FCs development. Numbers 1–5 on the branches represent different differentiation fate trajectories. (**B**) Trends in gene expression associated with branching in cells based on distinct differentiation fates. Significantly enriched GO terms in each cluster are shown on the right. M1-M5 represent gene modules 1–5. (**C**) Regulatory network of significant pathways involved in M1-M5. (**D**) Gene network associated regulation of *Runx2*. Circular nodes represent genes, and V-shaped nodes represent TFs.

Osteoblasts are bone-forming cells of mesenchymal origin and principal participants in bone cell biology, potentially specialized in maintaining skeletal integrity and sensing mechanical strain [87]. The pseudotime trajectory reveals the differentiation process of mesenchymal cells (MCs) into osteoblasts (OBs) (Branch 1) and fibrocytes (FCs) (Branch 2) (Figure 6A). The heatmap displays the expression profiles of the differentiation fate of differential genes associated with the two branches, categorized into five modules (M1–M5) (Figure 6B; Table S5). Genes in M1, M3, and M5 show high expression levels in cells located at the distal ends of both branches, while genes in modules M2 and M4 were mainly expressed in the pre-branch stages. During the differentiation of MCs into OBs (M1), they primarily participate in extracellular structure organization (GO:0043062) and regulation of lipase activity (GO:0060191). During the differentiation of mesenchymal cells (MCs) into fibrocytes (FCs) (M3 and M5), the main biological processes involved were neuron projection guidance (GO:0097485) and extracellular structure organization (GO:0043062). This suggests that these genes play significant roles in the differentiation of MCs into both OBs and FCs. Genes from M2 and M4 were highly expressed in pre-branch cells and were primarily enriched in extracellular matrix assembly (GO:00850299) and regulation of keratinocyte proliferation (GO:0010837). These genes may contribute to maintaining the undifferentiated state of MCs or preparing them for subsequent differentiation stages.

The KEGG pathway network analysis for M1–M5 showed that the PI3K-Akt signaling pathway exhibited the highest connectivity among all pathways (Figure 6C; Table S6). Numerous transcription factors (TFs) control osteoblasts differentiation. To identify TFs that may regulate the differentiation of cochlear osteoblasts, genes with the top 500 weight values and identified osteoblast TFs were selected to construct a regulatory network, illustrating their relationship (Figure S5A). Among these, we identified a crucial transcription factor, *Runx2*, which may play a significant role in the development of bat cochlear osteoblasts. Runt-related transcription factor 2 (*Runx2*) is expressed during the late stages of bone development and in chondroprogenitor cells [88]. It is one of the earliest markers of osteoblast differentiation [89]. As a key regulator of osteoblast formation, *Runx2* is involved in multiple steps of mammalian skeletal development. Among the genes regulated downstream of *Runx2* (Figure 6D), many are associated with osteoblast development, such as colony stimulating factor 1 (*CSF1*). *CSF1* is an osteoblast-derived factor that directly promotes osteoclast development and serves as a crucial regulator of bone resorption [78,87]. It may also play a key role in the maturation of osteoblasts and chondrocytes.

### 3.6. Localization of Deafness Genes and Positively Selected Auditory Genes in the Cochlea of Bats

Hearing loss (HL) affects approximately 1–3 out of every 1000 infants globally, severely impairing children’s communication and cognitive development. Hereditary factors account for 60–80% of these cases [90,91]. Hereditary hearing loss (HHL) is categorized into syndromic hearing loss (SHL) and non-syndromic hearing loss (NSHL) based on whether it is accompanied by symptoms in organs other than the auditory system. NSHL can be further subdivided by mode of inheritance: autosomal dominant deafness (DFNA), autosomal recessive deafness (DFNB), X chromosome linked, and mitochondrial [92]. HHL is the most common disabling sensorineural disease, with more than half of the affected patients resulting from genetic mutations and exhibiting significant genetic heterogeneity. To date, 153 genes have been identified as associated with NSHL (https://hereditaryhearingloss.org/, accessed on 6 May 2024), and approximately 300 genes have been linked to syndromic forms [42,93].

**Figure 7:**
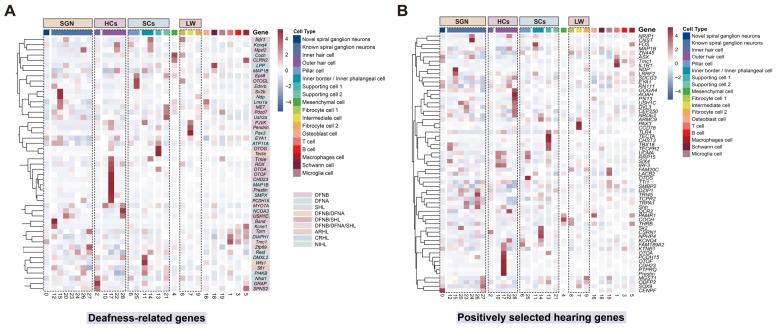
Cochlear cell expression patterns of deafness genes and positively selected hearing genes in bats. (**A**) Heatmaps of cell-type-specific expression (as a z-score for cell-type-averaged expression) for 49 deafness-related genes in cochlear cell clusters. DFNB: autosomal recessive deafness; DFNA: autosomal dominant deafness; SHL: syndromic hearing loss; ARHL: age-related hearing loss; CRHL: cisplatin-related hearing loss; NIHL: noise-induced hearing Loss. (**B**) Hierarchical clustering (as a *z*-score for cell-type-averaged expression) of cochlear cell types and 62 positively selected hearing genes (nonsynonymous to synonymous rate ratio (ω) > 1).

The retrieved deafness genes were mapped onto the single-cell atlas of cochlea in *H. armiger*, and detailed classification was performed according to cell type and deafness gene categories (Figure 7A). The majority of genes associated with HL were localized to HCs and SCs in the sensory epithelium, followed by SGN and SV cells. A total of 49 HL genes were identified, including 38 HHL genes, 32 NSHL genes (11 DFNA and 21 DFNB), 6 SHL genes, 2 DFNA/DFNB genes, 2 DFNB/SHL genes, and 1 DFNA/DFNB/SHL gene. Additionally, six NHL genes were identified, comprising three age-related hearing loss (ARHL), two cisplatin-related hearing loss (CRHL), and one noise-induced hearing loss (NIHL). Our analyses indicate that most of the genes mapped to bat cochlea are HHL genes, which are significant etiological factors in human hearing loss. The localization and expression of these genes in the bat cochlea may provide valuable insights for future studies on HL.

By integrating the positively selected and key hearing genes (62 genes) identified in recent years and mapping them onto the cochlea atlas of *H. armiger* (Figure 7B), we found that most of these genes are localized in HCs, SCs, and SGN cells. This suggests that HCs and SCs, due to their critical roles in auditory perception, are more susceptible to and prone to genetic mutations compared to other cell types within the cochlea. Notably, compared to humans, deafness genes and positively selected high-frequency hearing genes are also localized in SGN cells within the bat cochlea. These indicate that genetic factors may contribute to the adaptive changes in high-frequency hearing observed in bats.

## 4. Discussion

For nearly half a century, researches on bat high-frequency hearing were primarily focused on evolutionary biology [16,17,94,95,96,97,98], neurophysiology [99,100,101,102,103], and morphology [104,105,106,107,108,109,110,111,112,113], aiming to investigate the evolution of the inner ear’s neural anatomy, physiology, and morphology in bat species from multiple dimensions, and to further reveal the impact on echolocation. These multifaceted approaches seek to elucidate the physiological mechanisms underlying the evolution of high-frequency hearing in echolocating bats. With the development and utilization of scRNA-seq, researchers have been able to uncover the complex biological mechanisms of various cell types within auditory organs at the single-cell level, addressing the challenges of cellular heterogeneity that traditional bulk RNA-seq could not resolve. Although several pioneering studies have described the cellular composition of the cochlear sensory epithelium, sensory neurons, and SV in mice and birds at single-cell resolution [41,43,56,66,114,115], revealing the complexity and specificity of cochlear cell types, a comprehensive cell atlas for bat species with high-frequency hearing is still lacking. Furthermore, the precise localization of the identified deafness genes and positively selected genes within the bat cochlea remains to be elucidated.

### 4.1. New SGN Types and Their Potential Role in High-Frequency Hearing in H. armiger

In this study, we performed snRNA-seq analysis on the cochlea of *H. armiger* using both the PacBio-optimized genome [30] and the unoptimized original genome [31] as references. The results annotated with the PacBio-optimized genome showed superior performance in cell clustering compared to the original genome, particularly in the precise definition and clustering of SGN cell types in the cochlea. After optimization, the proportion of SGN cells in the total cochlear cell population increased by 45.20%, with most marker genes identified as novel genes from the optimized genome [30]. This highlights the effectiveness of the PacBio optimization, substantially improving the annotation of the original genome and enhancing the accuracy in defining the target clusters. Additionally, new types of SGN cells were identified in the cochlea of *H. armiger*. Re-clustering results of the SGN cells after optimization (Figure S2A–C) revealed that the new SGN types consist of three distinct subtypes, which may represent different variations. FISH validation confirmed that these neurons differ from the cochlear neurons previously reported in mammals, establishing them as new SGN cell types in the cochlea of *H. armiger*. These unique SGN subtypes could play a crucial role in echolocating bats, especially those with high-frequency hearing.

Echolocating bats, particularly CF-FM bats, developed a superior capacity to tune and precisely identify frequencies compared to humans and other animals. Behavioral studies have revealed that CF-FM bats employ the CF component of their echolocation signals to assess velocity-related information, while the FM component is used to interpret distance and detailed target information [18,116]. *H. armiger*, the targeted species in this study, is a typical CF-FM bat known for its exceptionally sharp frequency tuning. Throughout the entire ascending auditory pathway, from the cochlea to the auditory cortex, the CF component of its echolocation signal is overrepresented, forming an “auditory fovea”. Neurons in this region exhibit sharp frequency tuning curves to meet the needs of bats detecting Doppler shifts in target echoes. We hypothesize that the specific SGNs identified in this study may play a crucial role in Doppler shift compensation behavior in CF-FM bats, facilitating more efficient recognition and processing of acoustic signals associated with specialized behaviors. However, whether these special SGNs exist in the cochleae of other echolocating bats still need further studies to confirm.

Echolocating animals form images of their surroundings by comparing the sounds they emit with the echoes. Identification of the difference between the pulse sound signals and associated echoes is the central to echolocation. To realize this process effectively, the outgoing signal must be represented at the neuronal level for future comparison with the returning echoes. SGNs transmit information received from cochlear hair cells within the osseous spiral lamina. Data obtained from micro-computed tomography scans of 26 bat species demonstrated that the proximal end of the stylohyal, part of the mammalian hyoid apparatus, in connection with the tympanic bone, distinguishes laryngeal echolocating bats from non-echolocating bats, click bats, and other animals [117]. This connection between the stylohyal and the tympanic bone is one of the indicators of echolocation behavior in species [118], influencing the neuronal representation of outgoing signals and thus exhibiting differences at the neuronal level. The new types of SGN cells identified in bat cochlea in this study provide valuable insights for advanced research into high-frequency hearing.

### 4.2. Cochlear Cell Atlas of H. armiger

The first single-cell atlas of the cochlea in *H. armiger* was constructed, encompassing 16 cell types distributed across five major spatial locations. This provides a crucial piece for the whole map of molecular mechanisms illustrating high-frequency hearing in echolocating bats. Unlike birds and reptiles, mammals possess three auditory ossicles in their peripheral auditory systems. Currently, most single-cell transcriptomic studies of the mammalian cochlea focus on mice and humans [41,42,119,120], examining cells located inside and around the organ of Corti, as well as cells in the modiolus, SV, and spiral ligament/spiral limbus, along with various types of immune cells. These cell types are similar to those identified in this study. Combining the HE staining results of the cochlea in *H. armiger*, we found that the cochlear structure of bats is closely aligned with that of mice and humans, suggesting evolutionary similarities in the peripheral auditory structures among mammals.

In particular, we found a significantly higher number of immune cells in the cochlea of *H. armiger* compared to other mammals. This abundance of immune cells may serve to protect and repair auditory function under intense noise stimulation, ensuring sensitive high-frequency hearing. Echolocating bats have evolved in environments filled with intense noise, but in fact, their hearing remains healthy and functions well. Without such adaptations, their heavy reliance on hearing for echolocation would make survival impossible [44]. Using continuous intense noise stimulation to disrupt the synchronization between middle ear muscle activity and sonar vocalization [121], it was found that the cochlear hair cells of echolocating bats were more resistant to intense noise compared to non-echolocating bats and mice. This resilience was likely due to the adaptation of echolocating bats to noisy environments filled with intense sonar calls over their long-term evolution [44]. In addition to hair cells with immune functions (non-immune cells), the immune cells in the cochlea may also be indispensable. The heterogeneous composition of immune cell clusters and the significant increase in immune cell numbers in the cochlea following noise exposure may indicate the recruitment of innate and adaptive immune cells in the inner ear after noise-induced trauma [84]. In addition to the repair of auditory functions and protection of hair cells, the immune system could also function well in directly preventing infection of the cochlea and providing immunological protection. However, the specific mechanisms of immune cells in bat cochlea have not yet been elucidated, and further research is needed to explore these mechanisms in depth.

A comprehensive review of adaptive evolution genes identified in the high-frequency hearing perception of echolocating bats revealed 62 genes, including positively selected and key high-frequency hearing genes such as *Prestin*, *KCNQ4*, *Tmc1*, *CDH23*, *Shh*, and *Sk2*. Localization of these genes in the cochlear cell atlas showed that most are located in SCs, HCs, and SGNs, and to a lesser extent in lesser LW cells. SCs and HCs are core structures of the organ of Corti, whose primary function is to convert sound signals into electrical signals. The high proportion of positively selected genes in sensory epithelial suggests that SCs and HCs play a crucial role in the evolution of high-frequency hearing in bats and have undergone adaptive changes. Additionally, the localization of these genes in SGNs underscores the importance of neuronal transmission within the cochlea for the high-frequency hearing of echolocating bats. Therefore, the evolution of high-frequency hearing in bats hinges on the processes of sound signal conversion and transmission. In summary, this study identifies adaptive changes in these cell types as significantly contributing to the formation and refinement of high-frequency hearing in echolocating bats. Furthermore, it systematically reveals the key cell types involved in the development and evolution of high-frequency hearing, providing new insights into the origin and evolution of echolocation in mammals.

### 4.3. Potential Differentiating Relationship Between SCs and HCs

Non-mammalian animals, such as fish and amphibians, can regenerate sensory hair cells throughout their lives [122,123,124,125]. However, in mammals, the loss of sensory hair cells is typically irreversible, and the lack of hair cell regeneration capacity is a major cause of HL. Significant progress has been made in HC regeneration over the past few decades. Both mitotic and nonmitotic pathways have been identified to play important roles in HC regeneration. In the mitotic, progenitor cells re-enter the cell cycle and differentiate into new HCs [126]. In the non-mitotic, the molecular characteristics of SCs are altered, leading to their direct conversion into HCs [127,128,129]. Based on HE staining, the cochlear structure of *H. armiger* is similar with that of most mammals. The sensory epithelium is organized into one row of IHCs, three rows of OHCs, and interdigitating non-sensory SCs. PAGA and pseudotime trajectory analyses reveal differentiation relationships from SCs to OHCs and IHCs in the cochlea. Specifically, some SCs differentiate into IHCs, while others temporarily function as OHCs before fully transitioning into OHCs. However, the possibility of mitotic division forming new SCs, OHCs, and IHCs cannot be excluded, and further in-depth studies on cell differentiation are needed. Additionally, this study identified key genes involved in various differentiation fates, providing valuable references for research on hair cell differentiation and regeneration.

Studies have shown that SCs maintain a certain degree of plasticity, allowing them to convert into hair cell-like cells [130,131,132]. The mechanisms underlying this regenerative capability have begun to be elucidated [133]. Compared to organoids derived from older mouse cochleae (postnatal day 5), those from younger cochleae (postnatal day 2) form significantly more HCs [134,135]. The calcium signaling pathway is a key pathway enriched with differential genes involved in various differentiation fates. The dynamic balance of calcium between auditory HCs and the fluid they bathe in enables us to hear our first sound. Our interpretation and response to this sound requires rapid calcium flux through neuronal voltage-sensitive calcium channels [136]. Calcium-dependent action potentials have been shown to occur spontaneously in immature IHCs [137,138] but not in OHCs [139,140]. In the mammalian cochlea, immunohistochemistry and physiology indicate that IHCs contain submillimolar concentrations of Ca^2+^ binding sites. Surprisingly, the primary function of OHCs is mechanical amplification, with little role in sound encoding. They contain higher concentrations of Ca^2+^ buffers [141], which may play a crucial role in the stereocilia Ca^2+^ signaling and amplification processes in these cells [142]. Therefore, we propose that calcium signaling pathway may be one of the reasons for the different differentiation trajectories of SCs. The differentiation relationship and potential mechanisms between SCs and HCs in the bat cochlea could provide valuable references for future studies on hair cell regeneration.

### 4.4. Origin and Differentiation of Osteoblasts in Bat Cochlea

MCs are essential for the normal development and hearing of the cochlea [143,144,145]. MCs can terminally differentiate into various cell types, including specialized tympanic border cells, fibrocytes of the lateral wall and spiral limbus, basal cells of the stria vascularis, and modiolar osteoblasts [146]. The pseudotime trajectory indicates differentiation relationships from MCs to OBs and FCs. Differential fate genes were clustered into five modules based on their expression profiles, primarily involved in biological processes such as extracellular structure organization, neuron projection guidance, and extracellular matrix assembly. The PI3K-Akt signaling pathway and TGF-beta signaling pathway are the primary pathways enriched with differential fate genes generated during differentiation. Both pathways are widely recognized for their functions in osteoblast differentiation and bone formation, which are critical in mammalian skeletal development [147,148,149]. Bone homeostasis is maintained through a delicate balance between osteoblast-mediated bone formation and osteoclast-mediated bone resorption [78]. Relevant studies have found that positively selected genes in the echolocating bat cochlea are significantly enriched for processes related to ossification and the osteoclast differentiation pathway. Combined with the key pathways identified in this study, these findings indicate that both bone formation and osteoblast differentiation, critical for bone structure and function, have undergone adaptive changes in the echolocating bat cochlea [46].

Moreover, this study discovered a TF, *Runx2*, which may participate in the induction of osteoblast differentiation in the bat cochlea. *Runx2* is likely to regulate the potential and direction of early differentiation of bone marrow mesenchymal stem cells, thus influencing osteogenic differentiation. *Runx2* is not only a critical TF that regulates the initial stage of mesenchymal cell differentiation into osteoblasts and a specific terminal differentiation product; it also inhibits the maturation and proliferation of osteoblasts, preventing their further differentiation into osteocytes [150,151]. By upregulating the expression of PI3K subunits and Akt, *Runx2* enhances its DNA-binding ability in immature MCs, immature OBs, and prechondrocytes [152,153]. Among the genes regulated downstream of *Runx2*, many are associated with osteoblast development. For instance, *Runx2* and *CSF1* are marker genes that aid in identifying OBs and are key regulators of osteogenesis and bone resorption. *CSF1* has been found to be under positively selected in echolocating bats [46]. In brief, MCs in the bat cochlea possess the potency to differentiate into OBs and FCs. Genes and TFs related to different differentiation fates have undergone adaptive changes in mammals with high-frequency hearing.

### 4.5. New Perspective on Gene Targeted Therapy for Deafness

Hearing loss is a chronic condition affecting millions of people worldwide, with no current restorative treatment available. According to estimates by the World Health Organization, over 1.5 billion people are currently affected globally, and this number is projected to rise to 2.5 billion by 2050 [154] (http://www.who.int/features/factfiles/deafness/en/, accessed on 6 May 2024). Cochlear dysfunction and variations in specific genes can lead to a range of hearing impairments, placing the cochlea, a multicompartmental and complex structure, at the forefront of both fundamental biological research and translational neurobiology [115], especially concerning bat species with high-frequency hearing. Over half of the genes associated with deafness are expressed in the cochlea. Several groundbreaking studies have mapped these genes onto single-cell atlases of the mouse cochlea [41,42,43,155], aiming to identify cochlear target cell types for therapeutic applications at the cellular level. By integrating previous research and aggregated datasets, we mapped deafness genes onto the cellular atlas of the bat cochlea and identified HCs, SCs, SGNs, and SV as the primary cell types where these genes are located. Except for SGNs, the localization of human deafness genes in the mouse cochlea is largely consistent, with over 50% of the genes responsible for HHL being expressed in HCs. Published studies indicate that pathogenic genes in HCs, SCs, and SV are common targets for inner ear gene therapy [156]. Compared to humans, deafness genes are more frequently located in SGNs in the bat cochlea, suggesting the importance of SGNs for high-frequency hearing in bats. This is evidenced by the high incidence of mutations at deafness susceptibility loci within SGNs. Therefore, we propose that one of the reasons bats possess high-frequency hearing, unlike other mammals, is due to the role of their special SGN cells.

Despite the identification of over 150 deafness genes, there are currently no drugs available for clinical treatment [157]. For HL genes expressed in HCs, existing clinical treatments include cochlear implants or hearing aids, which bypass HCs and directly stimulate auditory nerve fibers, helping individuals with hearing impairments to regain auditory function [158,159]. The extensive localization of HL genes in SGNs highlights the importance of focusing on changes in neuronal cells within the cochlea to prevent hearing loss and other ear-related diseases. Studies have shown that *Rousettus aegyptiacus* experiences ARHL, or presbycusis, which deteriorates more severely at higher frequencies. Consequently, bats have also been considered a model organism for studying ARHL [160,161,162]. Overall, bats exhibit capabilities in cochlear repair, immune response, and hair cell regeneration. These traits offer valuable insights for the screening of deafness genes, targeted therapies, and the repair of hair cell damage across various types of HL, including genetic, noise-induced, and age-related deafness. Using bats as a model species for studying human HL holds potential medical value, helping to prevent and improve human HL. This research could open new avenues for the treatment of deafness and provide innovative ideas for the clinical application of cochlear implants.

## 5. Conclusions

In summary, by constructing a single-cell resolution transcriptome atlas of the cochlea using the PacBio-optimized genome as a reference, we annotated a total of 16 cell types and systematically revealed the molecular characteristics of cochlear cells in *H. armiger*. New types of SGN cells were identified, and the differentiation trajectories between different cells were revealed. Additionally, we clarified the cellular origins of positively selected genes associated with high-frequency hearing in bats and identified the susceptible cell types for human deafness genes. This study provides reliable data support for future research on the origins and adaptive evolution of high-frequency hearing in echolocating bats and offers valuable references for future studies on gene therapy for hearing loss.

## Data Availability

The raw sequence data reported in this article have been deposited in the Genome Sequence Archive at the National Genomics Data Center, China National Center for Bioinformation/Beijing Institute of Genomics, Chinese Academy of Sciences (GSA: CRA017915).

## Supplementary Materials

The following supporting information can be downloaded at https://www.mdpi.com/article/10.3390/biom15020211/s1, Figure S1: UMAP visualization of 27 clusters based on original genome; Figure S2: Re-cluster of the special SGNs; Figure S3: Expression of marker genes by cell type on a pseudotime trajectory; Figure S4: WGCNA analysis of each cell type; Figure S5: Regulatory network of TFs in osteoblasts; Table S1: Marker genes and expression for each cluster; Table S2: Single cell data utilization comparison between different references; Table S3: Differentiation fate differential genes in state 5234 vs. 51; Table S4: KEGG enrichment of differentiation fate differential genes in state5234 vs. 51; Table S5: Differentiation fate differential genes in state 21 vs. 2345; Table S6: KEGG enrichment of differentiation fate differential genes in state 21 vs. 2345.
