# Supplementary material for: Single-Cell Landscape of the Cochlea Revealed Cell-Type-Specific Diversification in Hipposideros armiger Based on PacBio Long-Read Sequencing
Source: Biomolecules. 2025 Feb 1;15(2):211. doi: 10.3390/biom15020211 (PMC11853400; doi:10.3390/biom15020211)
Supplement: Supplementary file 1 [file biomolecules-15-00211-s001.zip › biomolecules-3402521-Supplementary Figures S1-S5.pdf]

Single-Cell Landscape of the Cochlea Revealed Cell-Type-Specific Diversification in *Hipposideros armiger* Based on PacBio Long-Read Sequencing

Supplementary Figures S1-S5.

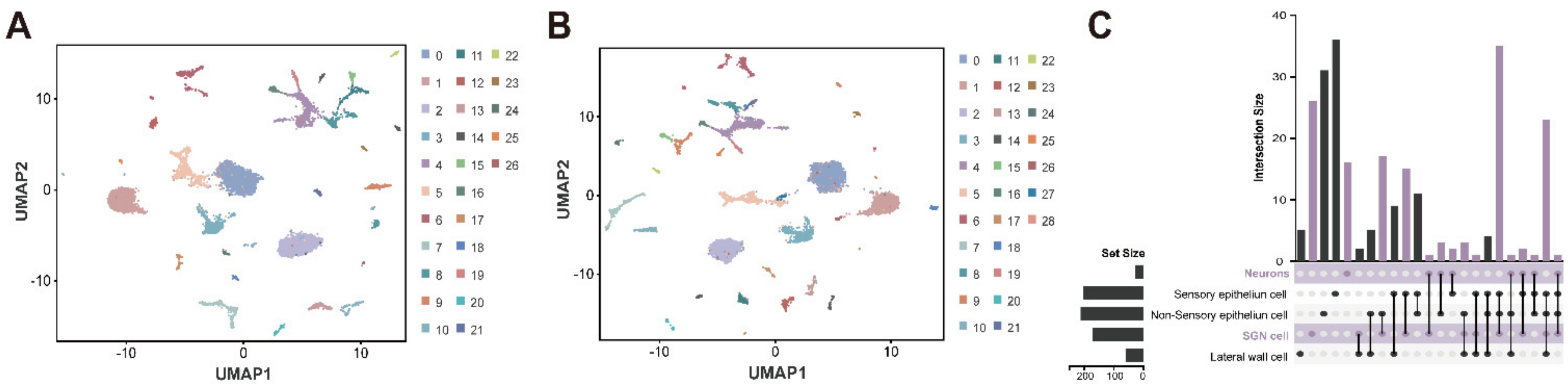

Figure S1. UMAP visualization of 27 clusters based on original genome. **A**) UMAP visualization of 27 cell clusters based on original genome. Each dot denotes a single cell. **B**) UMAP visualization of 29 cell clusters based on PacBio optimized genome. C) 2112 novel genes obtained after PacBio optimization were mapped to various cell types Upset maps.

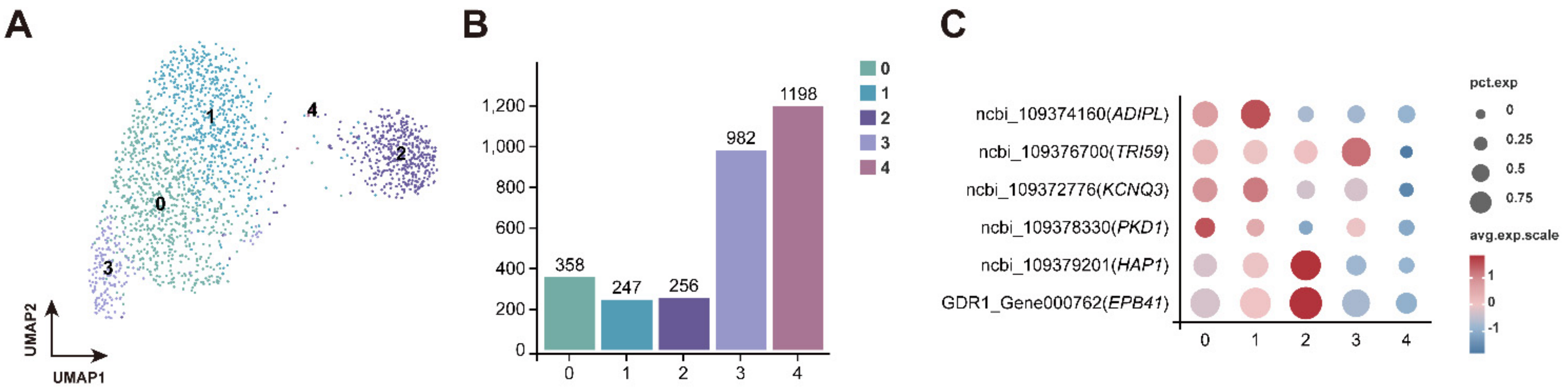

Figure S2. Re-cluster of the special SGNs. A) Novel SGN are divided into five subclusters. B) Number of up-regulated genes in five clusters. C) Bubble plot of marker genes for each cluster.

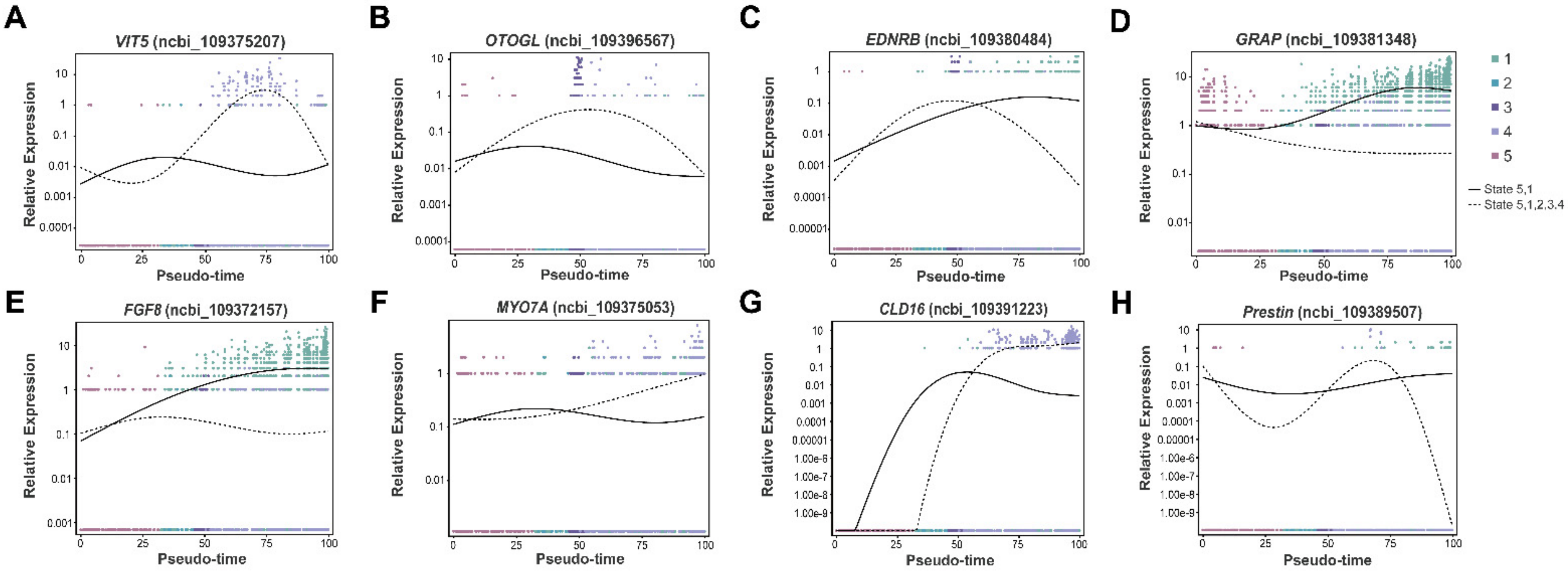

Figure S3. Expression of marker genes by cell type on a pseudotime trajectory. A-C) The scatter plot of marker genes differentiation fate of SCs shows that *VIT5*, *OTOGL*, and *EDNRB* are expressed in the middle segment of the initial branch and Branch1, mainly involved in cell development and HCs differentiation, representing the cell distribution of SCs on the pseudotime axis. D-E) Scatter plot of marker genes differentiation fate of IHCs, with *GRAP* and *FGF8* mainly expressed in Branch2, representing the cellular distribution of IHCs along the pseudotime axis. F-H) The scatter plot of marker genes differentiation fate of OHCs shows that *MYO7A*, *CLD16*, and *Prestin* are mainly expressed in the middle and end of Branch1, representing the distribution position of OHCs on the pseudotime axis. Colors indicate the level of gene expression in individual cells.

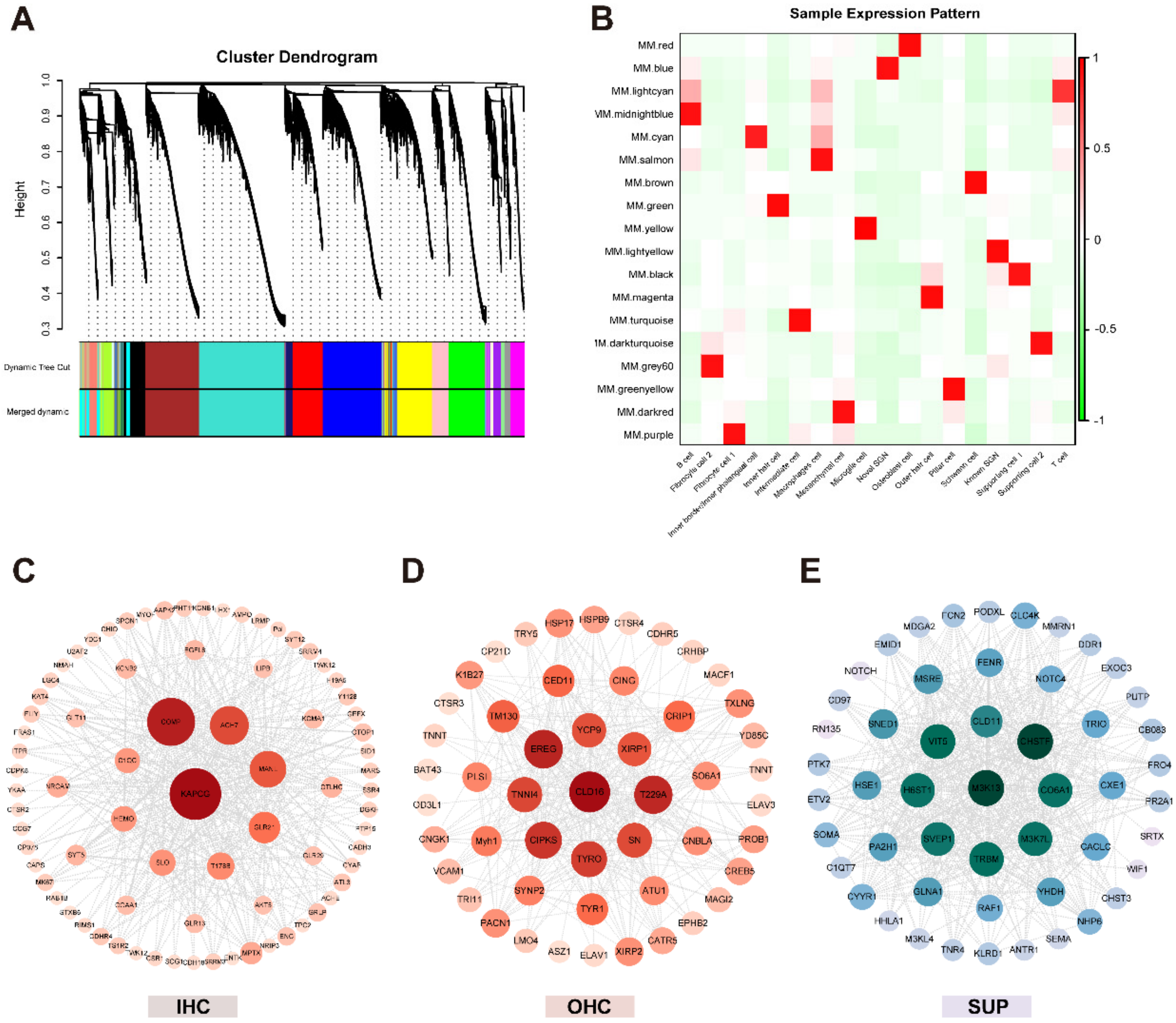

Figure S4. WGCNA analysis of each cell type. A) The diagram of module level clustering. B) Heatmap of different expression patterns of various cell types. C) Hub gene network screened by ranking the top 500 IHCs weight values. D) Hub gene network screened by ranking the top 500 OHCs weight values E) Hub gene network screened by ranking the top 500 SCs weight values.

A

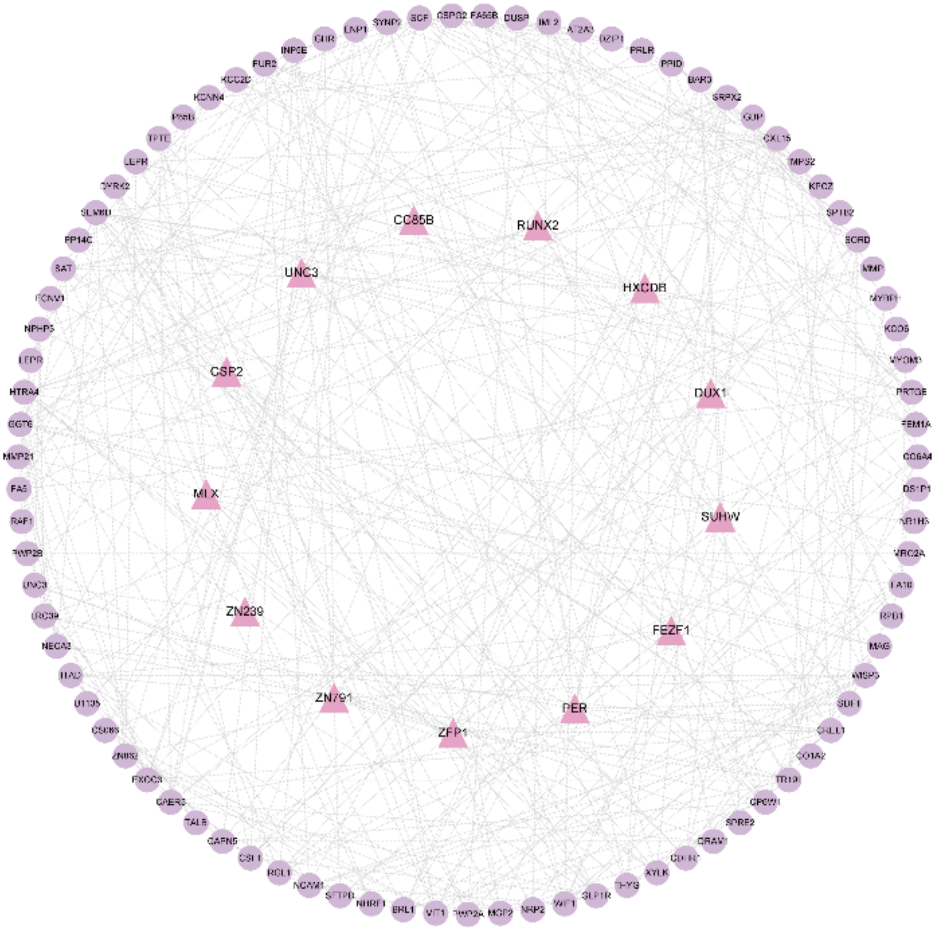

Figure S5. Regulatory network of TFs in osteoblasts. A) Network of gene transcription factor correlation in osteoblasts, with genes in the outer circle and transcription factors in the inner circle.
