# Supplementary material for: Single-Cell Landscape of the Cochlea Revealed Cell-Type-Specific Diversification in Hipposideros armiger Based on PacBio Long-Read Sequencing
Source: Biomolecules. 2025 Feb 1;15(2):211. doi: 10.3390/biom15020211 (PMC11853400; doi:10.3390/biom15020211)
Supplement: Supplementary file 1 [file biomolecules-15-00211-s001.zip › biomolecules-3402521-Supplementary Table S2.pdf]

Table S2 Single cell data utilization comparison between different references.

**Categories**	**Original genome (ncbi_GCF_001890085.1)**	**Genome optimized by PacBio (ncbi_SRR22307099)**
Number of Reads	405,139,884	405,139,884
Valid Barcodes	97.30%	97.30%
Sequencing Saturation	71.00%	71.10%
Q30 Bases in Barcode	95.70%	95.70%
Q30 Bases in RNA Read	89.20%	89.20%
Q30 Bases in UMI	92.70%	92.70%
Estimated Number of Cells	15,863	15,316
Number of Cells	11,112	11,265
Fraction Reads in Cells	53.30%	52.60%
Mean Reads per Cell	25,539	26,452
Median Genes per Cell	977	1,051
Total Genes Detected	20,418	20,949
Median UMI Counts per Cell	1,411	1,565
Reads Mapped Confidently to Genome	83.50%	83.60%
Reads Mapped Confidently to Intergenic Regions	13.60%	9.10%
Reads Mapped Confidently to Intronic Regions	37.80%	36.00%
Reads Mapped Confidently to Exonic Regions	32.10%	38.50%
Reads Mapped Confidently to Transcriptome	63.10%	67.50%
